# Investigating the Shelf-Life Extension of Shrimp Surimi Using a Polysaccharide-Based Film from *Alpinia oxyphylla*

**DOI:** 10.3390/foods15030530

**Published:** 2026-02-03

**Authors:** Meng Wang, Zengshuo Huang, Feng Li, Yebao Chen, Fangfang Ban, Hua Yang, Siming Zhu, Junlin Wu

**Affiliations:** 1 School of Food Science and Engineering, Guangdong Ocean University, 1 Luoqin, Yangjiang 529500, China; y13369321843@163.com (M.W.); 13430053111@163.com (Z.H.); lifeng0216gg@163.com (F.L.); chenyebao@gdou.edu.cn (Y.C.); banfangfang@126.com (F.B.); huayang@gdou.edu.cn (H.Y.); 2 School of Life and Geographic Sciences, Kashgar University, Kashi 844000, China; 3 School of Food Science and Technology, South China University of Technology, 381 Wushan Road, Tianhe District, Guangzhou 510640, China

**Keywords:** polysaccharide-based preservation film, sodium alginate, calcium ion cross-linking, shrimp surimi preservation

## Abstract

To investigate the effect of a polysaccharide-based composite film (ASC) composed of *Alpinia oxyphylla* polysaccharide (its molecular weight was approximately 4.07 kDa, and the monosaccharide composition was predominantly glucose and galacturonic acid), sodium alginate, and calcium chloride on the storage quality of shrimp surimi, this study compared the preservation efficacy of the ASC film with that of treatments using chitosan, potassium sorbate, ascorbic acid, sodium alginate, *Alpinia oxyphylla* polysaccharide, and distilled water. Samples were stored at 4 °C for 12 days, and evaluations were conducted by measuring film structural characteristics and quality indicators of shrimp surimi. Results showed that the ASC groups (where *Alpinia oxyphylla* polysaccharide was added at 20%, 30%, and 40% of the sodium alginate mass, designated as ASC 20%, ASC 30%, and ASC 40%) significantly outperformed the control group across all quality indicators. The ASC 30% group demonstrated the best overall preservation performance, effectively delaying oxidative browning, protein degradation, lipid oxidation, and microbial growth in shrimp surimi. The ASC 40% group exhibited particularly strong antibacterial effects, while the ASC 20% group also showed stable preservation performance. The composite film combines the antioxidant and antibacterial activities of *Alpinia oxyphylla* polysaccharide with the barrier and moisture-retention properties of sodium alginate, forming a stable three-dimensional network structure through calcium chloride cross-linking. It is superior to single/individual chemical preservatives in terms of film-forming ability, functionality, and safety, providing a natural, effective, and environmentally friendly preservation approach for shrimp surimi and other aquatic products. It also offers a theoretical foundation and practical reference for the development of natural preservation technologies in the food industry.

## 1. Introduction

Shrimp surimi, a paste made from minced and pounded fresh shrimp meat, is one of the most actively traded aquatic products in global seafood commerce. It exhibits vibrant color, a smooth and tender texture, and represents a high-market-value shrimp-based minced product [1,2]. With the rapid growth of the food industry, consumer expectations for food have increased significantly, particularly in terms of quality, texture, nutrition, flavor, and shelf life. Due to its pleasant taste, high protein content, and strong nutritional profile, shrimp surimi is widely favored by consumers. Growing demand has further solidified its important position in the market [3,4]. However, shrimp surimi’s high moisture and protein content also make it prone to spoilage during storage, as it is susceptible to degradation by microorganisms and enzymes. The deterioration process involves not only moisture loss but also protein and lipid oxidation, the formation of biogenic amines, and the development of off-odors. These changes ultimately reduce the product’s sensory quality and nutritional value while shortening its shelf life. To extend the shelf life of shrimp surimi, conventional preservation methods such as low-temperature refrigeration, modified-atmosphere packaging (MAP), and chemical preservatives are commonly used [5]. While MAP alters the gaseous environment around the product, its efficacy can be limited without a complementary high-barrier packaging material. The ASC film developed in this study is designed to provide such a barrier. Its dense, cross-linked polysaccharide network inherently possesses resistance to the permeation of oxygen and water vapor. This dual barrier function is crucial for maintaining a modified atmosphere and preventing quality deterioration, thereby offering a synergistic effect when combined with MAP technologies or serving as a stand-alone protective coating [6].

However, traditional preservation approaches often raise concerns regarding food safety, consumer health risks, and environmental pollution. Consequently, the development of natural, environmentally friendly, and safe preservation technologies has become a prominent research focus in the field of food preservation [7]. In recent years, natural polysaccharide-based composite films have garnered increasing attention due to their excellent preservation performance, safety, biodegradability, and environmental friendliness. For example, Feng Xuya et al. [8] prepared a chitosan-zinc oxide nanoparticle composite film. It effectively maintained stable meat color and improved sensory properties during the preservation of Tan sheep meat. Liu Hengli et al. [9] developed a novel composite edible coating. Using chitosan as the film-forming matrix and capsaicin as the antibacterial component, supplemented with glycerol and gelatin, this coating successfully extended the shelf life of pork. Additionally, Lü Rui et al. [10] fabricated a pectin-wheat gluten protein-sodium alginate composite film containing ε-polylysine. This film showed significant antibacterial and preservative effects, effectively inhibiting microbial growth, delaying lipid oxidation and protein decomposition, and maintaining the texture and water-holding capacity of snakehead fish fillets.

The fruit of *Alpinia oxyphylla*, recognized as one of the “Four Major Southern Medicinal Herbs,” is a medicinal and edible resource. It is known for regulating gut microbiota and enhancing immune function. Its polysaccharide component (AOFP) is a natural heteropolysaccharide [11]. Our extraction and characterization revealed that the primary polysaccharides are of the pectin type, rich in galacturonic acid. The molecular weight was approximately 4.07 kDa, and the monosaccharide composition was dominated by glucose and galacturonic acid. These polysaccharides contain abundant active groups, such as hydroxyl and carboxyl groups. These groups confer significant antioxidant and antibacterial activities [12], making AOFP a potential base material for preservation films. However, when used alone as a film-forming material, *Alpinia oxyphylla* polysaccharide shows relatively poor performance. It tends to form thin, fragile films with low mechanical strength and poor continuity. This is likely due to its relatively low molecular weight and insufficient chain length/linearity to form a robust, continuous matrix. Therefore, it needs to be combined with other robust film-forming materials [13]. Among various polysaccharides (e.g., starch, cellulose, pectin, chitosan), sodium alginate (SA) was selected for this study. SA was chosen for its superior film-forming ability, excellent oxygen barrier properties, high biocompatibility, and most importantly, its unique ability to undergo rapid, irreversible gelation. This gelation occurs via ionic cross-linking with divalent cations like Ca^2+^, forming a stable “egg-box” structure [14]. This property allows the formation of a cohesive, strong, and functional matrix that can effectively encapsulate and synergize with bioactive AOFP.

Sodium alginate, a natural linear polysaccharide extracted from algae, is widely used in the development of novel preservatives. When coated on the surface of food and dried, it forms a uniform and dense film that blocks the contact of external oxygen and microorganisms [15]. Additionally, it exhibits strong water-locking capacity and gelling properties, and can be used as a thickener to improve the cohesion of product structure, resulting in enhanced tensile strength, greater flexibility, and reduced breakage. Being biodegradable, it also aligns with modern environmental principles [15]. Calcium chloride, as a commonly used gelling cross-linking agent, is frequently employed in composite preservation films. The cross-linking mechanism predominantly follows the established “egg-box” model, where divalent Ca^2+^ ions cooperatively bind between the α-L-guluronate blocks of adjacent alginate chains, forming a stable three-dimensional network. This structure significantly enhances the film’s mechanical stability, water resistance, and barrier properties [16]. For instance, Liu Ziyi et al. used anhydrous calcium chloride as a cross-linking material to prepare sodium alginate porous materials based on this principle [17]. Calcium chloride, as a commonly used gelling cross-linking agent, is frequently employed in composite preservation films. The choice of Ca^2+^ over other divalent cations (e.g., Zn^2+^, Mg^2+^) was based on several factors. Ca^2+^ provides superior gel strength and stability due to its optimal charge density and ionic radius for the “egg-box” cavity. It also has Generally Recognized as Safe (GRAS) status for food applications, is cost-effective, and is well-established in alginate gelation literature [18].

Therefore, in this experiment, shrimp surimi was treated with the ASC composite film. It was then stored under refrigeration at 4 °C alongside samples treated with other common preservation methods, including chitosan solution, potassium sorbate solution, ascorbic acid solution, sodium alginate solution, *Alpinia oxyphylla* polysaccharide solution, and distilled water (control). Samples from each group were collected periodically to compare changes in appearance, color difference, pH, total volatile basic nitrogen (TVB-N), thiobarbituric acid reactive substances (TBARS/MDA), and total viable count (TVC). Furthermore, film characterization indicators such as Fourier-transform infrared spectroscopy (FTIR), scanning electron microscopy (SEM), and drying resistance were measured. The preservation effect of ASC on shrimp surimi was comprehensively analyzed based on the variation in these data, aiming to provide new theoretical support and practical evidence for the development of natural preservation technologies for shrimp surimi.

## 2. Materials and Methods

### 2.1. Experimental Materials

Fresh shrimp meat processed into shrimp surimi, Chaofudao brand (Manufacturer: Chaofudao Food Co., Ltd., City: Qingdao, Country: China); *Alpinia oxyphylla* fruit was purchased from Weiyuan Weiminyuan Biotechnology Co., Ltd. (Weiyuan, Gansu, China; batch number: 20241001); DEAE-cellulose 52 and dextran gel G-100 were supplied by Shanghai Yuanye Bio-Technology Co., Ltd. (Shanghai, China); n-Hexane, concentrated sulfuric acid, and phenol were obtained from Macklin Biochemical Technology Co., Ltd. (Shanghai, China); Sodium chloride, calcium chloride, anhydrous ethanol, chitosan, sodium alginate, and potassium sorbate were supplied by Sinopharm Chemical Reagent Co., Ltd. (Shanghai, China); Ascorbic acid was provided by Changde Beckman Biotechnology Co., Ltd. (Changde, Hunan, China); Glacial acetic acid was sourced from Tianjin Zhiyuan Chemical Reagent Co., Ltd. (Tianjin, China); Malondialdehyde (MDA) assay kit, total protein quantification assay kit, and total volatile basic nitrogen (TVB-N) assay kit were purchased from Nanjing Jiancheng Bioengineering Institute (Nanjing, Jiangsu, China).

### 2.2. Experimental Methods

#### 2.2.1. High-Temperature and High-Pressure Extraction of Polysaccharides

High-temperature and high-pressure extraction was performed according to a previously reported method [8]. Dried *Alpinia oxyphylla* was placed in an oven at 50 °C for 48 h until a constant weight was achieved. The material was then ground into powder using a grinder, and particles smaller than 150 μm (passing through a 60-mesh sieve) were collected and stored for further use. Five grams of the powder were mixed with distilled water at a ratio of 1:20 (*w*/*v*). After thorough mixing, the suspension was treated in an LDZF-50L high-pressure steam sterilizer at 121 °C and 0.1 MPa for 30 min, followed by cooling to room temperature. The extract was then centrifuged at 4000 rpm for 15 min, and the supernatant was collected and concentrated using rotary evaporation. To the concentrated solution, absolute ethanol was added to a final concentration of 80% (*v*/*v*). The mixture was kept at 4 °C for 12 h, and the precipitate was collected by vacuum filtration. The precipitate was washed three times with ethanol, and the solvent was evaporated to obtain the crude polysaccharide. The crude polysaccharide was redissolved in distilled water and deproteinized using the Sevag reagent (chloroform: n-butanol = 4:1, *v*/*v*) with shaking for 2 h. After centrifugation at 4000 rpm for 15 min, the upper protein layer was discarded. This deproteinization step was repeated 2–3 times. The deproteinized solution was dialyzed against distilled water using a dialysis membrane with a molecular weight cutoff of 1 kDa for 48 h. Finally, the dialyzed sample was further purified by sequential column chromatography on DEAE-52 cellulose and Sephadex G-100, followed by freeze-drying to obtain a white powder, designated as AOFP-HH.

#### 2.2.2. Determination of Molecular Weight and Monosaccharide Composition

The molecular weight of the polysaccharide was determined using a DAWN HELEOS II multi-angle laser light scattering detector coupled with an RID-20A refractive index detector, and a series of Shodex OHpak SB-806M HQ and SB-804M HQ columns (300 mm × 7.8 mm; Shodex, Tokyo, Japan) [19]. The column temperature was maintained at 40 °C, with 0.1 mol/L sodium nitrate solution (pH 5.5) as the mobile phase at a flow rate of 0.5 mL/min. For monosaccharide composition analysis, the polysaccharide sample was hydrolyzed with trifluoroacetic acid (TFA) at 121 °C for 2 h, followed by drying under a stream of nitrogen and washing three times with methanol [10]. The hydrolysate was filtered through a 0.22 μm membrane prior to analysis. Monosaccharide composition was analyzed using a Dionex ICS-5000^+^ system equipped with a Carbopac PA-20 column (4 mm × 250 mm; Thermo Fisher Scientific, Waltham, MA, USA)The mobile phase consisted of 97.5% ultrapure water containing 0.25 mmol/L NaOH and 2.5% 100 mmol/L NaOH solution, delivered at a flow rate of 1.0 mL/min, with the column temperature set at 30 °C. Qualitative and quantitative analyses were performed using standard solutions of D-mannose, D-glucose, D-galacturonic acid, and other monosaccharides.

#### 2.2.3. Preparation of *Alpinia oxyphylla* Polysaccharide-Sodium Alginate Composite Solutions

Three clean 250 mL beakers were each filled with 100 mL of distilled water. The water was heated to 70 °C in a water bath. Then, sodium alginate was added to each beaker at a concentration of 1% (*w*/*v*). Subsequently, *Alpinia oxyphylla* polysaccharide was added to the respective beakers. The AOFP addition levels were set at 20%, 30%, and 40% of the total mixed system. These specific concentrations were selected to investigate the film-forming performance while preventing issues arising from excessively high AOFP content or insufficient sodium alginate. The mixtures were stirred with a glass rod until completely dissolved. Finally, the solutions were cooled to room temperature and set aside for later use.

#### 2.2.4. Preparation of Anhydrous Calcium Chloride Solution and Verification of Rapid Film-Forming Properties

**Figure 1:**
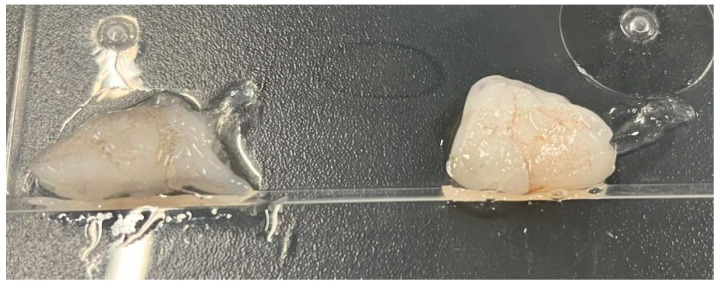
Verification of rapid film formation of anhydrous calcium chloride (Left: immersed in calcium chloride solution; Right: did not immerse in calcium chloride solution).

A clean 250 mL beaker was filled with 100 mL of distilled water. Then, 1% (*w*/*v*) calcium chloride was added and stirred until completely dissolved. The solution was set aside for later use. To verify the rapid film-forming capability of the system, fresh shrimp meat was cut into segments. One segment was immersed in a 2% sodium alginate solution for 5 min and then removed. Another segment received the same treatment but was subsequently immersed in the 1% calcium chloride solution for 5 s. Using tweezers to peel off the surface film, it was observed that the segment treated with calcium chloride formed a more intact, uniform, and tear-resistant film. This result confirmed the effectiveness of the ionic cross-linking step [20]. The rapid film-forming verification is illustrated in Figure 1.

#### 2.2.5. Treatment of Samples

Fresh shrimp meat was used as the raw material. The intestinal tract was removed, and the shrimp were thoroughly cleaned. The meat was then processed into a smooth and uniform surimi paste using a meat grinder. The paste was then portioned into blocks of equal weight using a standardized spoon to ensure consistent initial conditions across all experimental groups.

**Figure 2:**
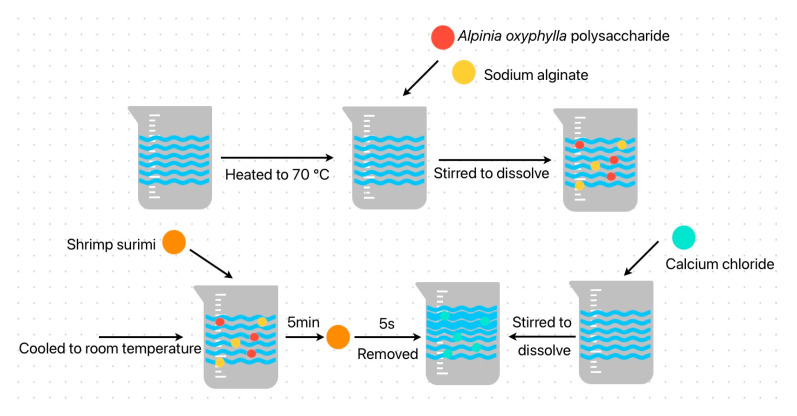
Schematic diagram of cross-linking preparation for ASC composite film. Note: The distilled water group served as a negative control to account for potential effects of the basic treatment steps (immersion and calcium ion cross-linking) themselves. Potassium sorbate and ascorbic acid were included as representative chemical preservatives (antimicrobial and antioxidant, respectively). They provided industry-standard benchmarks for evaluating the performance of the composite film. Chitosan and sodium alginate, as common biopolymer-based film-forming materials, were used to assess the preservation effects of single-component physical barrier coatings. These groups also served as controls for the corresponding components within the composite film. The *Alpinia oxyphylla* polysaccharide solution was applied alone to independently evaluate the contribution of the active polysaccharide, thereby distinguishing its functional role within the composite system. The three ASC composite film formulations were the core focus of this study. Their multifunctional and synergistic preservation performance was systematically evaluated through comparison with all the aforementioned control groups.

To enhance the adhesion and integrity of the coating film where calcium ion cross-linking significantly improves film performance the prepared shrimp surimi blocks were separately immersed for 5 min in the following solutions: distilled water (control group), 2% chitosan solution, 2% sodium alginate solution, 1% *Alpinia oxyphylla* polysaccharide solution, 0.1% potassium sorbate solution, 0.5% ascorbic acid solution, and three pre-selected composite solutions from Section 2.2.3. After achieving an even coating, the samples were removed and immediately immersed in a 1% CaCl_2_ solution for 30 s to induce cross-linking. They were then promptly taken out. All treated shrimp surimi samples were placed in sterile storage boxes and divided into four groups for refrigerated storage at 4 °C. Measurements were taken on days 0, 4, 8, and 12. Figure 2 shows the flow chart of ASC preparation [21].

#### 2.2.6. Determination of Thickness, Mechanical Properties, and Water Vapor Transmission Rate of CSAC

The thickness of the film was measured at five randomly selected positions on a flat, smooth area using a digital vernier caliper (model DHGDW150S; Delixi Electric Co., Ltd., Wenzhou, Zhejiang, China). For mechanical testing, film samples were cut into rectangular strips measuring 50 mm × 20 mm. Tensile strength and elongation at break were determined using a texture analyzer (model FTC-Pilot; FTC Corporation, Sterling, VA, USA). The water vapor transmission rate was measured using a gravimetric method. First, anhydrous calcium chloride was dried at 105 °C to a constant weight. A portion of the dried desiccant was then placed in a weighing cup, and the cup opening was sealed with a uniformly thick film sample, secured tightly with a rubber band. The initial weight was recorded. After 2 h, the assembly was weighed again. The rate was calculated from the weight gain. All measurements were performed in triplicate.

#### 2.2.7. Fourier-Transform Infrared (FTIR) Spectroscopy Analysis

Freeze-dried film samples (1–2 mg) from each group were mixed with spectroscopic-grade potassium bromide (KBr) at a mass ratio of 1:100 and ground homogenously in an agate mortar. The finely ground powder was compressed into transparent pellets using a hydraulic press. FTIR spectra were recorded on a Nicolet IS50 spectrometer (Thermo Fisher Scientific, Waltham, MA, USA) with a resolution of 4 cm^−1^ over the range of 4000–400 cm^−1^ and analyzed comprehensively [22].

#### 2.2.8. Scanning Electron Microscopy (SEM)

Freeze-dried films were mounted on metal stubs using conductive adhesive, sputter-coated with gold, and observed under an Apreo 2S high-resolution field-emission scanning electron microscope (Thermo Fisher Scientific, Waltham, MA, USA) at appropriate accelerating voltage and current. Images were captured at magnifications of 100× and 1000× for morphological analysis [23,24].

#### 2.2.9. Anti-Drying Test

The prepared coating solutions of each group were poured into glass Petri dishes (triplicate per group) and placed in a naturally ventilated area. Weight loss was recorded daily for 5 consecutive days.

#### 2.2.10. Appearance Evaluation

Treated shrimp surimi samples of each group were stored at 4°C. Visual changes were photographed and observed on days 0, 4, 8, and 12 over the 12-day storage period.

#### 2.2.11. Color Measurement

Surface color of shrimp surimi was measured using a colorimeter (CR-400, Guangzhou Yousheng Instrument Co., Ltd., Guangzhou, China) on days 0, 4, 8, and 12. Values of *L* (lightness), *a* (red-green), b* (yellow-blue), and the total color difference (Δ*E*) were recorded. Measurements were taken at 2–3 points on each sample surface. Δ*E* was calculated as:

$${∆E}^{*}=\sqrt{{\left({L12}^{*}-{L0}^{*}\right)}^{2}\;+{\left({a12}^{*}-{a0}^{*}\right)}^{2}+{\left({b12}^{*}-{b0}^{*}\right)}^{2}}$$

where subscript 0 denotes day 0 and subscript 12 denotes day 12.

#### 2.2.12. pH Measurement

pH was determined following GB 5009.237-2016 [25] “National Food Safety Standard- Determination of pH in Food” using a pH meter (FE28; Mettler Toledo, Shanghai, China). A representative sample (1 g) was homogenized with 10 mL distilled water using a high-speed disperser (T25 easy clean digital; IKA, Staufen, Germany). The electrode was immersed in the homogenate, and the stable reading was recorded. Triplicate measurements were averaged for each sample.

#### 2.2.13. Total Volatile Basic Nitrogen (TVB-N)

TVB-N content was measured with a commercial assay kit according to the manufacturer’s instructions.

#### 2.2.14. Malondialdehyde (MDA) Content

The MDA concentration was determined using a commercial MDA assay kit (A003-1) manufactured by the Nanjing Jiancheng Bioengineering Institute (Nanjing, China), following the provided protocol.

#### 2.2.15. Total Viable Count (TVC)

TVC was performed according to GB 4789.2-2022 “National Food Safety Standard- Microbiological Examination of Food-Determination of Total Bacterial Count.” Samples were collected on days 0, 4, 8, and 12. After aseptic pretreatment, serial dilutions were prepared with sterile saline. An appropriate dilution (200 µL) was spread onto Plate Count Agar (PCA) in triplicate, alongside a blank control. Plates were incubated at 36 °C for 48 h, colonies numbering 30–300 were counted, and results were expressed as log_10_ CFU g^−1^ [26].

### 2.3. Data Processing and Statistical Analysis

Statistical analysis was performed using SPSS 22.0 software (IBM, Armonk, NY, USA), and data are presented as the mean ± standard error (n = 3, biological replicates). Graphs were plotted using Origin 2024 (OriginLab, Northampton, MA, USA). For comparisons among different treatment groups at the same storage time, one-way analysis of variance (ANOVA) was applied, followed by Tukey’s honestly significant difference (HSD) post hoc test when significant differences were detected (*p* < 0.05). Differences between groups are indicated by different lowercase letters (*p* < 0.05).

## 3. Results

### 3.1. Monosaccharide Composition and Molecular Weight

The yield of AOFP-HH obtained from the extraction was 13.65 ± 0.41%. As shown in Table 1, the monosaccharide composition was primarily composed of glucose and galacturonic acid. Molecular weight, a key physicochemical parameter that influences polysaccharide bioactivity and functional properties, was determined to be approximately 4.07 kDa for the homogeneous AOFP-HH fraction [16]. These structural characteristics are closely related to its potential biological activity and provide fundamental data for elucidating its structure-activity relationship. Furthermore, the *Alpinia oxyphylla* polysaccharide used in this study exhibited no undesirable or aromatic odors, making it particularly suitable for food preservation applications.

### 3.2. Analysis of Infrared Spectroscopy Data

**Figure 3:**
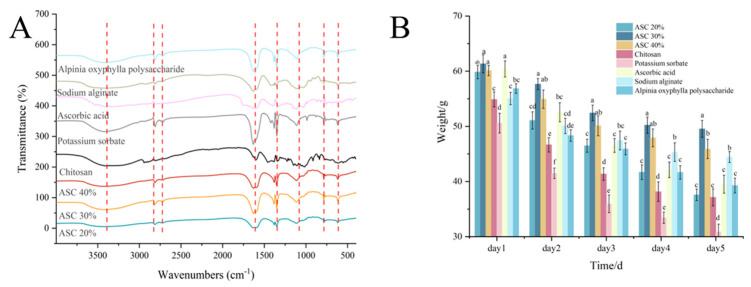
(**A**): FTIR Analysis; (**B**): Anti drying property results. Different lowercase letters at the same time indicate significant differences between groups (*p* < 0.05).

The FTIR spectra of the prepared films are presented in Figure 3A. All ASC film formulations (20%, 30%, and 40%) exhibited a broad and strong absorption band in the 3500–3300 cm^−1^ region, corresponding to O–H stretching vibrations. Compared to the spectrum of pure sodium alginate (SA), this band was broader and more intense in the ASC films, suggesting the formation of an extensive intermolecular hydrogen-bonding network between AOFP and SA [27]. In the spectrum of pure SA, the asymmetric and symmetric stretching vibrations of the –COO^−^ groups appeared at approximately 1600 cm^−1^ and 1418 cm^−1^, respectively. In all ASC composite films, these two characteristic peaks shifted to lower wavenumbers. This consistent negative shift confirms the coordination of Ca^2+^ ions with the carboxylate oxygen atoms. The interaction increases electron density in the C–O bonds, lowering their stretching frequency and providing direct spectroscopic evidence of successful ionic cross-linking. Additional bands in the regions of 2800–2400 cm^−1^ (C–H stretching) and 1200–1000 cm^−1^ (C–O–C and C–O stretching) indicate that the fundamental carbohydrate backbone structures of both polysaccharides remain intact within the composite films [28]. Collectively, the presence, positions, and shifts of these characteristic absorption bands verify the successful incorporation of AOFP and SA into the film matrix. They also demonstrate synergistic interactions between the components, involving both hydrogen bonding and ionic cross-linking via the egg-box model.

### 3.3. Anti-Drying Analysis

As shown in Figure 3B, the anti-drying properties of the different preservatives were evaluated by monitoring weight changes over five consecutive days. All treatment groups exhibited a gradual decline in weight over time, reflecting the loss of moisture during storage. The ASC 30% group demonstrated the most stable and optimal performance, with a significantly (*p* < 0.05) lower weight loss than the second-best group from day 2 onward, highlighting its superior water-retention capacity. The ASC 40% group ranked second, while the ASC 20% group showed results comparable to the SA-alone group. The weight-loss curve of the ASC 30% group displayed the gentlest slope, indicating sustained and balanced moisture retention. Moreover, the relatively small error bars associated with its data points reflect low variability and good reproducibility across the triplicate experiments, confirming the reliability of its performance. In contrast, although several groups initially showed similar weight loss, they diverged noticeably in later stages, with final weight-loss amplitudes exceeding those of the ASC films. The potassium sorbate group consistently exhibited the poorest water-retention ability throughout the experimental period.

The superior and sustained anti-drying performance of the ASC films, particularly the ASC 30% formulation, can be attributed to the synergistic effects of its components. Sodium alginate forms a dense, hydrophilic network that physically hinders moisture evaporation. Its abundant hydroxyl and carboxyl groups also establish strong hydrogen bonds with water molecules, effectively immobilizing free water. The incorporation of *Alpinia oxyphylla* polysaccharide, which itself is rich in hydrophilic hydroxyl groups, further enhances this water-binding capacity. Crucially, cross-linking mediated by Ca^2+^ ions bridges the guluronate blocks of adjacent alginate chains via the “egg-box” model, significantly strengthening the film’s three-dimensional structure. This robust and cohesive network not only improves mechanical integrity but also reduces the matrix’s free volume and permeability, thereby slowing the diffusion and loss of water during storage. The optimal balance of components in ASC 30% likely creates a microstructure that maximizes this multi-faceted water-retention mechanism.

In summary, the ASC 30% formulation significantly inhibits the drying of coated shrimp surimi during storage. Due to the molecular mechanisms described above, it exhibits sustained and stable effectiveness, demonstrating optimal preservation performance and application potential.

### 3.4. Scanning Electron Microscopy Analysis

**Figure 4:**
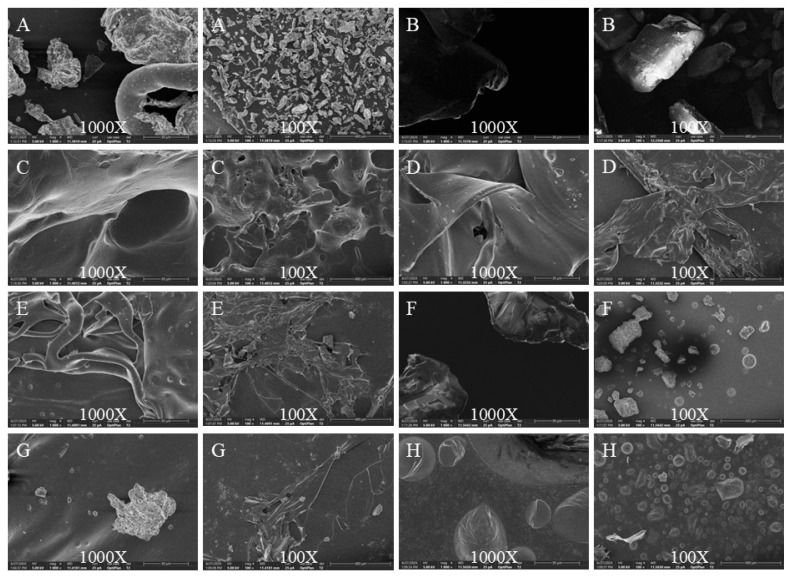
Scanning Electron Microscopy. A scale bar of 1000× corresponds to 30 μm, and a scale bar of 100× corresponds to 400 μm. (**A**) ASC 20%. (**B**) ASC 30%. (**C**) ASC 40%. (**D**) Chitosan. (**E**) Potassium Sorbate. (**F**) Ascorbic Acid. (**G**) Sodium Alginate. (**H**) *Alpinia oxyphylla* fructus polysaccharides.

The surface morphology of the films was examined using SEM (Figure 4). All ASC films (20%, 30%, and 40%) formed continuous, cohesive layers without major cracks or fragmentation, confirming successful film formation. Their microstructures, however, varied with AOFP concentration. The ASC 20% film displayed a slightly looser structure with relatively larger and more isolated pores. In contrast, the ASC 30% film showed the most uniform and integrated morphology, characterized by a coherent and interconnected porous network. This connectivity suggests the formation of continuous channels within the film matrix, which is a critical morphological feature that could facilitate the diffusion and controlled release of the antimicrobial AOFP agent. The ASC 40% film appeared denser, with fewer and smaller visible pores. Control films exhibited structural limitations. While chitosan and sodium alginate films showed some film-forming ability, their structures were less homogeneous. Films derived from potassium sorbate, ascorbic acid, or pure AOFP were either fragmented, loose, or brittle, failing to form cohesive layers. These morphological differences suggest that the concentration of AOFP influences the microstructure of the composite film. A balanced concentration (30%) appears to optimize the interactions (e.g., hydrogen bonding and ionic cross-linking) with the alginate matrix, facilitating the formation of a more integrated and percolating network that contributes to the film’s physical integrity and potentially modulates the release profile of active components.

### 3.5. Thickness, Mechanical Properties, and Water Vapor Transmission Rate of ASC

The properties of the ASC composite film are closely related to its compositional ratio. As shown in Table 2, systematic variations in film performance were observed as the proportion of *Alpinia oxyphylla* polysaccharide increased from 20% to 40%, reflecting the complex interplay between polysaccharide blending and the cross-linked network structure.

Film thickness gradually decreased from 0.17 mm (ASC 20%) to 0.12 mm (ASC 40%). This trend may be attributed to the rheological behavior of the film-forming solution [6]. Although the proportion of AOFP increases, the relative concentration and cross-linking density of sodium alginate as the continuous phase likely dominate the final shrinkage and densification process of the film, leading to a slight reduction in thickness as the AOFP content increases.

Tensile strength decreased significantly from 90.04 MPa (ASC 20%) to 78.5 MPa (ASC 40%). This suggests that a high proportion of AOFP (e.g., 40%) may partially disrupt the well-ordered, robust “egg-box” cross-linked network formed between sodium alginate chains and calcium ions [18]. While interpenetration of AOFP chains can introduce additional physical entanglement points, it may also reduce the uniformity and density of ionic cross-links, thereby lowering the material’s load-bearing capacity [8]. The elongation at break improves steadily from 45% to 53.44%. This is a clear positive indicator, demonstrating that the incorporation of AOFP effectively enhances the film’s flexibility and toughness. As another type of biopolysaccharide, AOFP provides additional molecular chain slippage and extensibility, partially counteracting the brittleness of the pure sodium alginate-calcium cross-linked network, enabling the material to withstand greater deformation before fracture [13]. The water vapor transmission rate increases from 1.64 g/(m^2^·h) to 2.25 g/(m^2^·h). This clearly indicates that as the proportion of hydrophilic AOFP rises, the film’s moisture barrier performance declines.

### 3.6. Changes in Appearance of Shrimp Surimi

**Figure 5:**
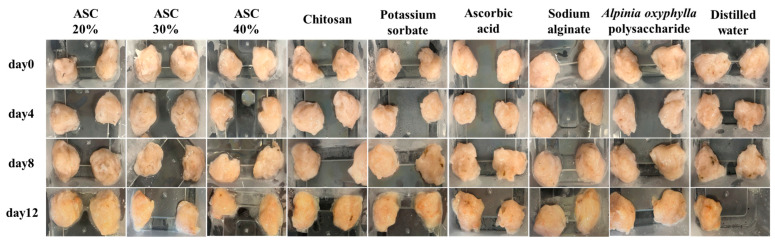
Changes in the appearance of shrimp surimi in different treatment groups.

Figure 5 illustrates the visual changes in shrimp surimi during storage. The ASC 30% group exhibited the best preservation effect, showing the least degree of yellowing and darkening throughout the storage period. On day 12, its color remained noticeably brighter than that of other groups. From day 0 to day 8, shrimp surimi treated with ASC 30% maintained a relatively light, original color with only moderate yellowing and darkening. The ASC 20% and ASC 40% groups, which contain AOFP at 20% and 40% of the SA mass, respectively, showed similar antioxidant and barrier properties to ASC 30%. Consequently, their color changes followed a comparable trend, though discoloration progressed slightly faster. Chitosan and sodium alginate, as single-polysaccharide preservatives, can form a basic physical barrier but lack antioxidant activity. By day 8, samples in these groups already showed noticeable yellowing and loss of brightness, with a similar extent of color change between the two.

Ascorbic acid provides antioxidant protection but no physical barrier. Although it delayed discoloration initially, substantial microbial proliferation in later stages accelerated pigment degradation. *Alpinia oxyphylla* polysaccharide alone offers antibacterial properties but has limited antioxidant capacity; therefore, surimi treated with this component also showed pronounced yellowing and darkening by day 8. Potassium sorbate showed only a limited preservative effect, while the distilled-water control provided no protection. In the control group, rapid oxidation of myoglobin and carotenoids, combined with the production of colored microbial metabolites, led to obvious yellowing and dullness as early as day 4, representing the poorest performance among all treatments.

Although the ASC series demonstrated better efficacy in delaying color deterioration, by day 8, all groups had developed off-odors, and some exhibited black spots. By day 12, all shrimp surimi samples showed severe yellowing and darkening due to protein denaturation and cumulative microbial metabolites. The texture became soft, collapsed, and hollow, indicating spoilage and rendering the product inedible.

### 3.7. Effect of Various Preservatives on the Color Difference in Shrimp Surimi

**Figure 6:**
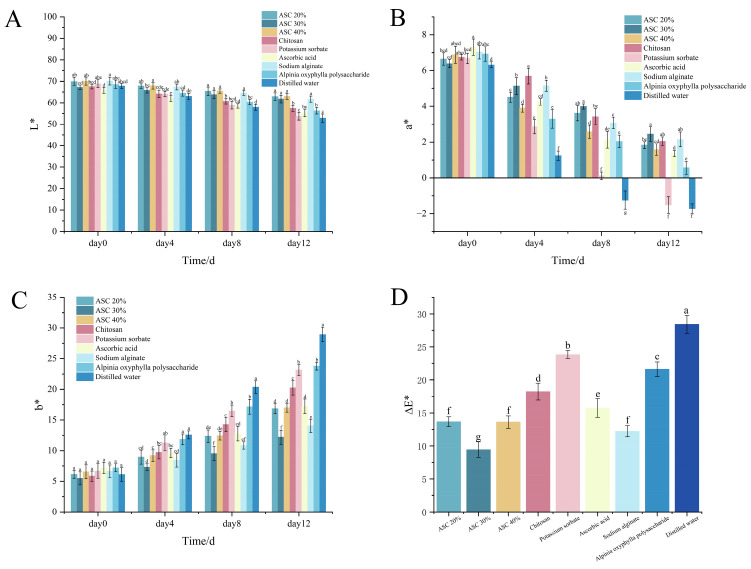
Effects of different treatments (ASC, Chitosan, Potassium Sorbate, Ascorbic Acid, Sodium Alginate, *Alpinia oxyphylla* fructus polysaccharides) on Smooth Color Difference. (**A**) L* Value of shrimp surimi. (**B**) Effects of different treatments on Smooth Color Difference a* Value of shrimp surimi. (**C**) Effects of different treatments on Smooth Color Difference b* Value of shrimp surimi. (**D**) Effects of different treatments on Total Color Difference ∆E* of shrimp surimi. Different lowercase letters at the same time indicate significant differences between groups (*p* < 0.05).

Figure 6 presents the changes in colorimetric parameters for each treatment group. The *L* value, indicating lightness, ranges from 100 (white) to 0 (black). As shown in Figure 6A, the *L* values of all treated groups decreased over time, reflecting a decline in brightness associated with quality deterioration. The ASC 30% group exhibited the smallest reduction in *L*, demonstrating the best preservation effect. On day 0, all groups showed similar *L* values with no significant differences. As storage progressed, the ASC 30% group maintained the highest lightness, reaching 61.73 even on day 12. In comparison, the *L* values on day 12 for the chitosan, potassium sorbate, ascorbic acid, *Alpinia oxyphylla* polysaccharide, and distilled water groups were 57.35, 53.73, 55.23, 56.24, and 52.84, respectively. The rate of decrease in *L* was slowest in the ASC 30% group, which was significantly lower than that of the other single-preservative groups. Although no significant difference was observed among the ASC 20%, ASC 30%, and ASC 40% groups, the ASC 30% group showed the smallest decline with lower variability, indicating its superior ability to inhibit darkening and maintain lightness in shrimp surimi.

In colorimetric measurement, the *a* value indicates the red–green axis of color, with higher positive values correlating with a redder hue. In the context of meat and seafood, this typically reflects less myoglobin oxidation, which is associated with better color retention and preservation quality. According to Figure 6B, *a* values decreased in all groups during storage. The ASC 30% group showed the smallest decline, indicating the best color retention. Initially, all groups had similar a values. By day 4, the *a* value of the ASC 30% group remained at 5.13, while those of the distilled water and potassium sorbate groups dropped to 1.24 and 2.86, respectively, highlighting the stronger initial redness retention of ASC 30%. By day 12, the distilled water and potassium sorbate groups showed pronounced redness deterioration. The *a* values of the ASC 20% and ASC 40% groups decreased to 1.83 and 1.58, respectively, whereas the ASC 30% group retained an *a* value of 2.45, demonstrating significantly better redness stability.

The *b* value reflects the yellow-blue tendency; a higher *b* value indicates more yellowness. As shown in Figure 6C, b values increased over time in all groups. The ASC 30% group exhibited the smallest increase, confirming its superior preservation effect. Throughout storage, the b values of ASC 30% were the lowest among all groups, recorded as 5.49, 7.32, 9.54, and 12.18 on days 0, 4, 8, and 12, respectively. The total increase over 12 days was only 6.69, which was substantially lower and more gradual than that of other groups. Moreover, significant differences (*p* < 0.05) were observed between ASC 30% and other groups on days 8 and 12, confirming that its yellowness development was markedly slower.

The total color difference (Δ*E*) indicates overall color stability, with lower values corresponding to a more stable appearance and color [29]. As illustrated in Figure 6D, the ΔE of the ASC 30% group was significantly lower than that of other groups, demonstrating the highest color stability over the 12-day storage period. This benefit arises from the combined effects of the antioxidant properties of *Alpinia oxyphylla* polysaccharide, the barrier function of sodium alginate, and the cross-linking contributed by calcium chloride in the composite film. Furthermore, the 30% concentration achieved the optimal balance, more effectively delaying oxidative browning and quality deterioration in shrimp surimi [30]. The ASC 20%, ASC 40%, chitosan, sodium alginate, *Alpinia oxyphylla* polysaccharide, and ascorbic acid groups all showed some stabilizing effect, while the distilled water and potassium sorbate groups exhibited significantly higher Δ*E* values, indicating the greatest color change and the poorest preservation performance.

### 3.8. Effect of Various Preservatives on the pH Value of Shrimp Surimi

**Figure 7:**
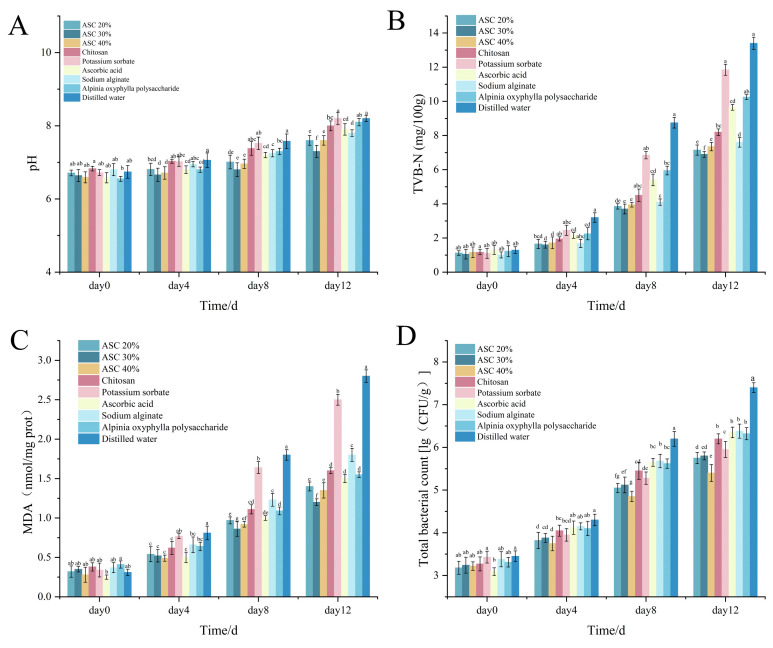
(**A**) Effect on pH value of shrimp surimi. (**B**) Effect on TVB-N value of shrimp surimi. (**C**) Effect on MDA value of shrimp surimi. (**D**) Effect on total bacterial count of shrimp surimi. Different lowercase letters at the same time indicate significant differences between groups (*p* < 0.05).

According to Figure 7A, the pH of shrimp surimi in all treatment groups increased progressively during the 12-day storage period, remaining relatively stable in the early stage and rising more rapidly later. The pH of the ASC 30% group consistently remained the lowest throughout storage, reaching only 7.3 on day 12, with a total increase of merely 0.66—far lower than that of the distilled water group and other preservative treatments. Moreover, the pH values of the ASC 30% group on days 4, 8, and 12 were significantly lower (*p* < 0.05) than those of the distilled water, potassium sorbate, *Alpinia oxyphylla* polysaccharide, and other single-preservative groups, indicating the strongest inhibition of pH rise during spoilage. The ASC 20%, ASC 40%, chitosan, and sodium alginate groups also exhibited significantly lower pH values than the distilled water and potassium sorbate groups in the later storage stage, confirming that these treatments provided measurable preservation effects. Given the established correlation between pH increase and spoilage in shrimp surimi, the low magnitude and slow rate of pH rise in the ASC 30% group demonstrate its superior ability to suppress microbial proliferation and protein degradation. Therefore, the ASC 30% treatment showed the best overall preservation performance. Nevertheless, by the end of storage, the pH of nearly all samples had reached a level indicative of unacceptably reduced freshness, rendering them unsuitable for consumption. This indicates that, although the preservative treatments were effective, they cannot extend the shelf life indefinitely.

### 3.9. Effect of Various Preservatives on the Total Volatile Basic Nitrogen (TVB-N) Content of Shrimp Surimi

Figure 7B illustrates the changes in TVB-N content of shrimp surimi treated with different preservatives over 12 days. TVB-N levels increased in all groups during storage, which is consistent with reported trends in aquatic products [31]. The increase was gradual in the early stage but accelerated markedly later. The ASC groups (20%, 30%, and 40%) consistently maintained the lowest and most comparable TVB-N values. On day 0, all groups exhibited similar TVB-N contents, reflecting the initial freshness of the shrimp surimi. By day 4, TVB-N values had increased moderately across groups, with the distilled water group showing a significantly faster rise. On days 8 and 12, the TVB-N values for the ASC groups were significantly lower than those of other single-component treatments (e.g., chitosan, AOFP alone) and chemical preservatives. Notably, after 12 days of storage, the sodium alginate (SA)-treated group also exhibited a relatively low TVB-N value, which was comparable to those of the ASC film groups. In contrast, the potassium sorbate and distilled water groups reached much higher values at the same time points, indicating severe spoilage.

These results demonstrate the superior efficacy of the ASC groups in stabilizing TVB-N levels, confirming that the ASC composite film effectively inhibits protein degradation and microbial proliferation in shrimp surimi. The comparable performance of the SA group to the ASC films at the later storage stage suggests that the dense, cross-linked sodium alginate matrix plays a primary and fundamental role by providing a sustained physical barrier against oxygen and microbes. However, the consistently superior performance of the ASC groups, especially ASC 30%, throughout the entire storage period highlights a critical synergistic effect. The incorporated *Alpinia oxyphylla* polysaccharide contributes active antioxidant and antimicrobial functions, which complement the passive barrier property of SA. This synergy leads to more effective inhibition of protein decomposition and microbial activity, thereby further reducing the production of volatile basic nitrogen. Therefore, the preservation performance of ASC films is attributed not to a single dominant component but to the action where SA forms a stable protective matrix and AOFP provides active biochemical inhibition.

### 3.10. Effect of Various Preservatives on the Malondialdehyde (MDA) Content of Shrimp Surimi

As shown in Figure 7C, the malondialdehyde (MDA) content in shrimp surimi treated with various preservatives increased during storage. A moderate rise was observed in all groups by day 4, with no significant differences among treatments. By day 8, except for the ASC groups, all other groups exhibited a sharper and more pronounced increase in MDA, a trend consistent with previous studies on lipid oxidation in aquatic products [32]. Throughout storage, the ASC 30% group maintained the lowest MDA level, reaching only 1.20 nmol mg^−1^ protein on day 12, with the most gradual increase over time. This indicates that the composite film containing *Alpinia oxyphylla* polysaccharide, sodium alginate, and calcium chloride at this concentration achieved an optimal balance between physical barrier properties and the release of active components, thereby most effectively limiting oxygen permeation and inhibiting oxidation. In comparison, the final MDA values for ASC 20% and ASC 40% were slightly higher, at 1.40 and 1.35 nmol mg^−1^ protein, respectively. This suggests that a lower polysaccharide content may result in an insufficient barrier or antioxidant capacity, whereas a higher concentration may compromise film uniformity and adhesion, slightly reducing overall efficacy. On day 12, the MDA contents for ascorbic acid, chitosan, and *Alpinia oxyphylla* polysaccharide alone were 1.5, 1.6, and 1.55 nmol mg^−1^ protein, respectively. While these single-component treatments also exhibited antioxidant activity, their performance was inferior to that of the ASC composite film. Potassium sorbate, a chemical preservative, showed relatively moderate antioxidant capacity, reaching 2.5 nmol mg^−1^ protein by the end of storage. The distilled water (control) group exhibited the highest MDA level at 2.8 nmol mg^−1^ protein. Collectively, these results demonstrate that the ASC 30% film significantly outperforms other treatments in inhibiting lipid oxidation and reducing MDA formation in shrimp surimi, confirming its superior preservation effect.

### 3.11. Effect of Various Preservatives on the Total Bacterial Count of Shrimp Surimi

Total viable count (TVC) is a comprehensive indicator reflecting the extent of microbial contamination and proliferation in aquatic products. Shrimp surimi, rich in protein and moisture, provides a nutrient-rich substrate for psychrotrophic spoilage bacteria such as *Pseudomonas*. Initially, fresh shrimp surimi exhibits a low TVC. As storage time progresses, microorganisms multiply extensively, decomposing nutrients and producing spoilage metabolites, leading to a sharp increase in TVC [33]. This microbial growth correlates with the development of off-odors, a sticky and loose texture, and other sensory deteriorations, accompanied by potential food safety risks. According to GB 10136-2015 “National Food Safety Standard for Animal Aquatic Products,” a TVC exceeding 5 log CFU/g is considered non-compliant.

As shown in Figure 7D, the TVC of shrimp surimi treated with all preservatives increased over the 12-day storage period. In the initial stage (days 0 and 4), TVC values for all groups remained below the national standard limit, indicating acceptable freshness. By day 8, most groups had exceeded this limit and were deemed unsuitable for consumption. On day 12, the ASC 40% treatment group showed the lowest TVC at 5.40 log CFU/g, demonstrating a significantly stronger bacteriostatic effect than all other groups. The final TVC values for ASC 20% and ASC 30% were similar. The potassium sorbate treatment group, as a chemical preservative, recorded 5.95 log CFU/g on day 12, showing the second-best performance, followed by the chitosan group. In contrast, ascorbic acid, sodium alginate, and *Alpinia oxyphylla* polysaccharide groups all showed relatively high and similar TVC values on day 12, suggesting that as single agents relying primarily on antioxidant or basic film-forming properties, their antimicrobial effects were limited. The distilled water control group, with no preservative function, exhibited the highest TVC increase, reaching 7.4 log CFU/g.

The growth trend of TVC across all groups was relatively slow in the early stage, likely due to the combined effects of preservatives and refrigeration [34]. Growth accelerated in the mid-storage period, indicating the onset of the logarithmic growth phase for microorganisms. The most rapid increase occurred in the later stage, reflecting substantial decomposition of the shrimp surimi matrix and a sharp acceleration of spoilage. These results further highlight the effectiveness of the ASC 40% treatment in maintaining the lowest microbial load during extended storage.

Despite the observed bacteriostatic effects, the final TVC values in all groups were notably elevated, indicating that shrimp surimi entered a state of advanced spoilage by the end of the storage period. This suggests that the edible period of shrimp surimi under 4 °C refrigeration is relatively short, approximately 4–5 days, and prolonged storage is not recommended.

This study relied solely on TVC to assess microbial spoilage and did not identify the specific dominant spoilage microorganisms. While TVC provides a useful general indicator of microbial growth, the absence of taxonomic data limits a detailed mechanistic interpretation of the antimicrobial effects observed. Future work incorporating microbial community analysis would help clarify the specific inhibitory actions of the preservative treatments.

## 4. Discussion

This study investigated the quality deterioration of shrimp surimi during storage and explored a preservation strategy using a composite polysaccharide-based film. The spoilage process involves multiple mechanisms. Spoilage bacteria secrete proteases that degrade myofibrillar proteins, disrupting the protein network [35]. This leads to reduced water-holding capacity, loss of elasticity, and textural defects such as softening, collapse, and loss of surface gloss. Simultaneously, enzymatic browning contributes to color darkening [36]. Microbial and endogenous enzyme activities also decompose proteins, generating alkaline nitrogenous compounds that increase pH and total volatile basic nitrogen (TVB-N) levels, often accompanied by off-odors [37,38]. Furthermore, the oxidation of unsaturated fatty acids produces malondialdehyde, a key indicator of lipid oxidation [38]. Rapid microbial growth can cause the total viable count to exceed safety limits, posing a direct food-safety risk [39]. Therefore, developing preservation technologies that address these multiple deterioration pathways is essential.

To extend the shelf life of shrimp surimi, this study employed a homogeneous polysaccharide extracted from *Alpinia oxyphylla* fruit (AOFP-HH) as a functional base material. ASC composite preservation films were prepared at different concentrations by cross-linking AOFP-HH with sodium alginate and calcium chloride. The abundant hydroxyl groups in AOFP-HH enable extensive hydrogen bonding, while its galacturonic acid residues facilitate ionic cross-linking with Ca^2+^. Together, these interactions establish a stable structural foundation for the composite film.

Structural characterization confirmed the successful fabrication of the film and revealed structure-function relationships. Fourier-transform infrared spectroscopy showed a broadened O–H stretching band (3500–3300 cm^−1^) in the ASC film, indicating the formation of a dense hydrogen-bond network between AOFP and SA. This network underpins the film’s excellent water-retention capacity. Scanning electron microscopy further showed that the ASC 30% film possesses a uniform, porous, network-like microstructure, constructed through hydrogen bonding, hydrophobic interactions, and Ca^2+^-mediated ionic cross-linking. This interconnected porous network acts as a continuous, dense physical barrier, effectively blocking oxygen and microorganism penetration while enabling the controlled release of active components. In contrast, the ASC 20% film exhibited a looser structure due to insufficient AOFP content, and the ASC 40% film appeared overly dense with reduced porosity, likely due to excessive polysaccharide. These observations indicate that a 30% AOFP concentration achieves an optimal balance between barrier function and active-component release.

Based on its structural features, the ASC film exerts preservation effects through a triple mechanism encompassing physical barrier, antioxidant activity, and antibacterial action. First, its dense three-dimensional network limits oxygen permeation, thereby delaying lipid oxidation and protein degradation. Its intrinsic UV-absorption capacity also provides protection against light-induced damage. Second, the film’s strong water-retention ability reduces moisture loss during storage, helping maintain the texture and sensory quality of shrimp surimi. Finally, bioactive components from AOFP-HH confer antioxidant and antibacterial properties. Phenolic hydroxyl and other functional groups can scavenge free radicals and inhibit lipid peroxidation chains, while the antibacterial activity directly suppresses microbial growth [40].

It is noteworthy that the ASC 30% film performed best in maintaining color and inhibiting chemical deterioration (e.g., pH, TVB-N, and MDA), whereas the ASC 40% film showed only a slight advantage in antibacterial efficacy. This suggests that at 20% AOFP, the synergy between active components and the film matrix is insufficient. At 40%, although the antibacterial compound content is higher, the excessively dense structure may hinder the release and effective contact of active components with the shrimp surimi, thereby impairing functions such as antioxidant activity. These findings highlight that the performance of the composite film is not a simple sum of its components, but rather a result of careful tuning among film structure, release kinetics, and functional requirements.

## 5. Conclusions

This study demonstrates that the composite preservation film, ASC—composed of *Alpinia oxyphylla* polysaccharide, sodium alginate, and calcium chloride—significantly preserves the quality of refrigerated shrimp surimi. All three ASC formulations (20%, 30%, and 40% AOFP relative to SA mass) outperformed single-component treatments (chitosan, potassium sorbate, ascorbic acid, SA alone, AOFP alone) and the distilled-water control across key quality indicators, including color difference, pH, total volatile basic nitrogen, malondialdehyde content, and total viable count. These results indicate that the ASC film, by forming a physical barrier and providing antioxidant and antibacterial activity, effectively delays oxidative browning, protein degradation, lipid peroxidation, and microbial proliferation in shrimp surimi. Among the ASC groups, ASC 30% exhibited the most balanced performance, characterized by an optimal film density, controlled release of active components, and superior water-retention capacity. ASC 20% and ASC 40% also displayed distinct advantages—the former in structural stability and the latter in antibacterial efficacy. The preservation mechanism arises from the synergy between AOFP (antioxidant/antibacterial), SA (oxygen barrier and moisture retention), and CaCl_2_ (ionic cross-linking). This combination forms a stable, interpenetrating three-dimensional network, which is evidenced in FTIR spectra by a denser hydrogen-bond network and in SEM images by a continuous, intact film morphology. Additionally, the film exhibits excellent anti-drying properties. Compared to chemical preservatives, ASC is safer, leaves no residue, and aligns with environmental standards. Relative to single-polysaccharide films, it offers enhanced film-forming ability and multifunctionality. Thus, ASC provides a safe, effective, and eco-friendly preservation strategy for shrimp surimi and other aquatic products, offering both theoretical insights and practical support for the development of natural food-preservation technologies.

## Figures and Tables

**Table 1:** Results of monosaccharide composition, molecular weight, and extraction yield of AOFP HH.

Monosaccharide Composition	Monosaccharide Composition	Monosaccharide Composition	Monosaccharide Composition
Mannose	3.1965	4.07 kDa	13.65 ± 0.41%
Galacturonic acid	11.7405		
Glucose	67.3359		
Galactose	5.7209		
Xylose	5.3015		
Arabinose	6.7048		

**Table 2:** Thickness, mechanical properties, and water vapor transmission rate of ASC.

Group	Thickness (mm)	Tensile Strength (MPa)	Elongation at Break (%)	Water Vapor Transmission Rate (g·mm·m^2^·h^−1^·Kpa^−1^)
ASC 20%	0.17 ± 0.09	90.04 ± 2.45	45 ± 1.3	1.64 ± 0.04
ASC 30%	0.13 ± 0.08	85.2 ± 3.10	48.57 ± 2.0	1.95 ± 0.05
ASC 40%	0.12 ± 0.10	78.5 ± 3.80	53.44 ± 2.8	2.25 ± 0.04

## Data Availability

The original contributions presented in this study are included in the article. Further inquiries can be directed to the corresponding authors.
